# Predicting postoperative gastric cancer prognosis based on inflammatory factors and machine learning technology

**DOI:** 10.1186/s12911-023-02150-2

**Published:** 2023-03-31

**Authors:** Cheng-Mao Zhou, Ying Wang, Jian-Jun Yang, Yu Zhu

**Affiliations:** 1 Big data and artificial intelligence research group, Department of Anaesthesiology, Central People’s Hospital of Zhanjiang, Zhanjiang, Guangdong China; 2 Department of Anesthesiology, Pain and Perioperative Medicine, First Affiliated Hospital of Zhengzhou University, Zhengzhou, Henan China

**Keywords:** Machine learning, Gastric cancer, Postoperative, Death, Inflammatory

## Abstract

**Objective:**

There is a strong association between gastric cancer and inflammatory factors. Many studies have shown that machine learning can predict cancer patients’ prognosis. However, there has been no study on predicting gastric cancer death based on machine learning using related inflammatory factor variables.

**Methods:**

Six machine learning algorithms are applied to predict total gastric cancer death after surgery.

**Results:**

The Gradient Boosting Machine (GBM) algorithm factors accounting for the prognosis weight outcome show that the three most important factors are neutrophil-lymphocyte ratio (NLR), platelet lymphocyte ratio (PLR) and age. The total postoperative death model showed that among patients with gastric cancer from the predictive test group: The highest accuracy was LR (0.759), followed by the GBM algorithm (0.733). For the six algorithms, the AUC values, from high to low, were LR, GBM, GBDT, forest, Tr and Xgbc. Among the six algorithms, Logistic had the highest precision (precision = 0.736), followed by the GBM algorithm (precision = 0.660). Among the six algorithms, GBM had the highest recall rate (recall = 0.667).

**Conclusion:**

Postoperative mortality from gastric cancer can be predicted based on machine learning.

## Introduction

Gastric cancer (GC) is a malignancy that originates from gastric epithelial cells. Its histological type is mostly adenocarcinoma. It can be classified as either early gastric cancer or advanced gastric carcinoma, based on stage at diagnosis. Early gastric cancer refers to cases where the cancer cell invasion depth is located in the gastric mucosa or submucosa. Regardless of the surface area of the lesion and lymph node metastasis, once the cancer cell invasion depth exceeds the submucosa, it is considered advanced gastric carcinoma. As the fifth most common malignant tumor in the world, more than 70% of the cases of gastric cancer occur in developing countries. About half of them occur in East Asia, mostly in China. Gastric cancer is the third leading cause of cancer death among men and women all over the world (behind lung and liver cancer), while its mortality rate is the highest in East Asia (14.0 men and 9.80 women per 100,000) [1]. Studies have shown that most patients have a higher 5-year survival rate after treatment by endoscopic mucosal resection and endoscopic submucosal dissection in the early stages [2]. However, in the advanced stages, the 5-year survival rate is low, even after surgery and chemotherapy [3]. Prognosis prediction and treatment plan selection based on traditional TNM Classification of Malignant Tumors staging and histological typing are the most widely used methods in clinical practice at present. However, even though some patients may have the same TNM staging and treatment plans, their prognosis could still be very different. Preoperative identification of patients at high risk of death will allow clinicians to perform early intervention and select those patients most likely to benefit from new therapies such as immunization for individualized treatment, thereby improving the prognosis. Therefore, there is an urgent need to improve the existing prediction models and establish new models that can accurately judge prognosis and guide treatment selection.

Inflammation is a key process in the occurrence and progression of malignant tumors. In recent years, more has been learned about the relationship between inflammation and cancer [4, 5]. Many cancers are induced by chronic inflammation [6, 7]. When there are persistent chronic inflammatory reactions, pathogens cannot be eliminated by inherent immunity or adaptive immunity. Therefore, a reaction occurs, which induces tumor cell production. The tumor growth destroys the tissue structure and produces inflammatory factors. This produces an array of inflammatory cells which hinder the regression of inflammation, thus promoting tumor growth and transmission [13]. Many studies have shown that neutrophil-lymphocyte ratio (NLR) and platelet lymphocyte ratio (PLR) reflect patients’ inflammation and immune status, and that they are prognostic factors of multiple tumors (including rectal, prostate, lung, and breast cancer) [8–10].

Artificial intelligence (AI) is an emerging technical science that simulates, extends and expands the theory, methodology, technology and application systems of human intelligence. In the contemporary medical apparatus and instrument field, most artificial intelligence collects data through a machine. It then optimizes and analyzes the data, ultimately leading to either a qualitative or quantitative solution. For instance, machine learning has been used to predict mortality associated with complications after radical cystectomy for bladder cancer [11]. Machine learning based on a series of cancer antigen 125 levels can also predict the recurrence of abdominal and pelvic cancer via CT scan[12]. Furthermore, machine learning has been used to predict early biochemical recurrence after robot-assisted prostatectomy [13]. However, there have been no related studies on mortality prediction for gastric cancer, after surgery. Therefore, this study addresses this gap in the scientific literature.

## Materials and Methods

### Study population

The present study is a secondary analysis. Data is available on the BioStudies database (https://www.ebi.ac.uk/biostudies/studies?query=S-EPMC5373584). The study involved 1,056 GC patients who had undergone gastrectomy.

According to postoperative histological specimens, all patients had been diagnosed with Stage I-III gastric carcinoma by histology. Tumor staging was performed using the seventh edition of the American Joint Cancer Commission (AJCC) TNM staging system [14]. Criteria for exclusion and inclusion were: (1) there had been no neoadjuvant chemotherapy or radiotherapy; (2) there was complete clinical pathology and follow-up data on potential prognostic factors; (3) there was no recurrence of gastric cancer, residual gastric cancer or other synchronous malignant tumors; (4) there was no acute infection or other inflammation within two weeks before surgery.

The following data were collected: age, sex, preoperative routine laboratory examination, post-operative tumor characteristics and survival time. Blood samples were collected a week before surgery. Papilla and moderately differentiated GC were divided into highly differentiated groups. Signet-ring cell, mucinous and undifferentiated GC were divided into mildly differentiated groups [15].

### Biomarker calculation

NLR and PLR were defined as absolute neutrophil and platelet counts divided by absolute lymphocyte count [16]. According to previous studies, COP-NLR was calculated as follows: patients with elevated platelet counts (> 300 × 10^9^/ L) and neutrophil-lymphocyte ratios (> 3) were scored as 2. Those without abnormal values were scored as either 1 or 0 [17].

Main outcome: Follow-up was conducted every 3 months for the first 2 years after surgery, and every 6 months thereafter. The overall state of death is defined as all causes of death after surgery.

### Machine learning algorithm

Logistic Regression (LR) is a supervised classification algorithm. For classification, the target variable (or output) *y* can only adopt discrete values for a given set of features (or inputs) *x*. Logistic regression establishes a regression model to predict the probability that a given data input can be classified into a category numbered “1”. As linear regression hypothesis data follows a linear function, logistic regression uses a sigmoid function to model the data.

Decision tree algorithms (Tr) are a type of supervised learning which can solve regression and classification problems. The decision tree uses tree representation to solve this problems in which each leaf node corresponds to a class label, and its attributes are represented on the internal nodes of the tree.

Based on the integration of Bagging (Bagging with the self-service sampling method) based on decision tree, random forest (forest) introduces random attribute selection into the decision tree’s training process. In a random forest, for each node of the base decision tree, a subset containing the *k* attributes is randomly selected from the node’s attribute set. Then, an optimal attribute is selected from the subset.

Gradient Boosting Decision Tree (GBDT) is a Boost algorithm (Boosting is a class of algorithms that promote weak learners over strong learners). It can also be considered an improvement of the Boost algorithm. Every calculation of it will reduce the residual of the previous one. In order to reduce these residuals, a new model can be established in the direction of a gradient with a reduced residual.

As a fast, distributed and high performance gradient-lifting framework based on the decision tree algorithm, Gradient Boosting Machine (GBM) can sort, classify, run regressions, and perform many other machine learning tasks.

Extreme gradient Boosting (Xgbc) incorporates both boosting algorithms and a lifting tree model, which integrates many tree models.

Data processing method: Data analysis was conducted in R. Preoperative inflammatory indexes for the two groups, and quantitative data such as NLR and PLR, were expressed by mean value ± standard deviation for independent t-tests. The difference was considered statistically significant at P < 0.05. The correlation analysis was conducted by python, and the machine learning algorithm was analyzed, while the prognosis weight was constructed with the LightGBM algorithm. The data were randomly divided into a training group and a testing group at a 7:3 ratio. In this study, five cross-validations were used.

## Results

Comparison of basic indexes between the two groups: There was no statistical significance between the two groups (P = 0.862). PLR and NLR in the poor prognosis group were greater than those of the control group, and there was statistical significance between the two groups (P < 0.05) (see Table 1**)**.

**Table 1:** Descriptive statistics on patients

Death	No	Yes	P-value
N	806	524	
Age (years)	56.1 ± 12.1	59.9 ± 11.2	< 0.001
Tumor size (cm)	4.3 ± 2.5	5.2 ± 2.6	< 0.001
NLR	2.3 ± 1.6	3.0 ± 3.4	< 0.001
PLR	145.0 ± 71.6	156.8 ± 93.5	0.033
Sex			0.862
Female	259 (32.1%)	166 (31.7%)	
Male	547 (67.9%)	358 (68.3%)	
Tumor location			< 0.001
Upper third	253 (31.4%)	258 (49.2%)	
Middle third	173 (21.5%)	105 (20.0%)	
Lower third	380 (47.1%)	161 (30.7%)	
Histological grade			0.005
Well differentiated	155 (19.2%)	70 (13.4%)	
Poorly differentiated	651 (80.8%)	454 (86.6%)	
TNM stage			< 0.001
1	203 (25.2%)	17 (3.2%)	
2	267 (33.1%)	67 (12.8%)	
3	336 (41.7%)	440 (84.0%)	
COP.NLR			< 0.001
0	537 (66.6%)	293 (55.9%)	
1	222 (27.5%)	192 (36.6%)	
2	47 (5.8%)	39 (7.4%)	

**Fig. 1:**
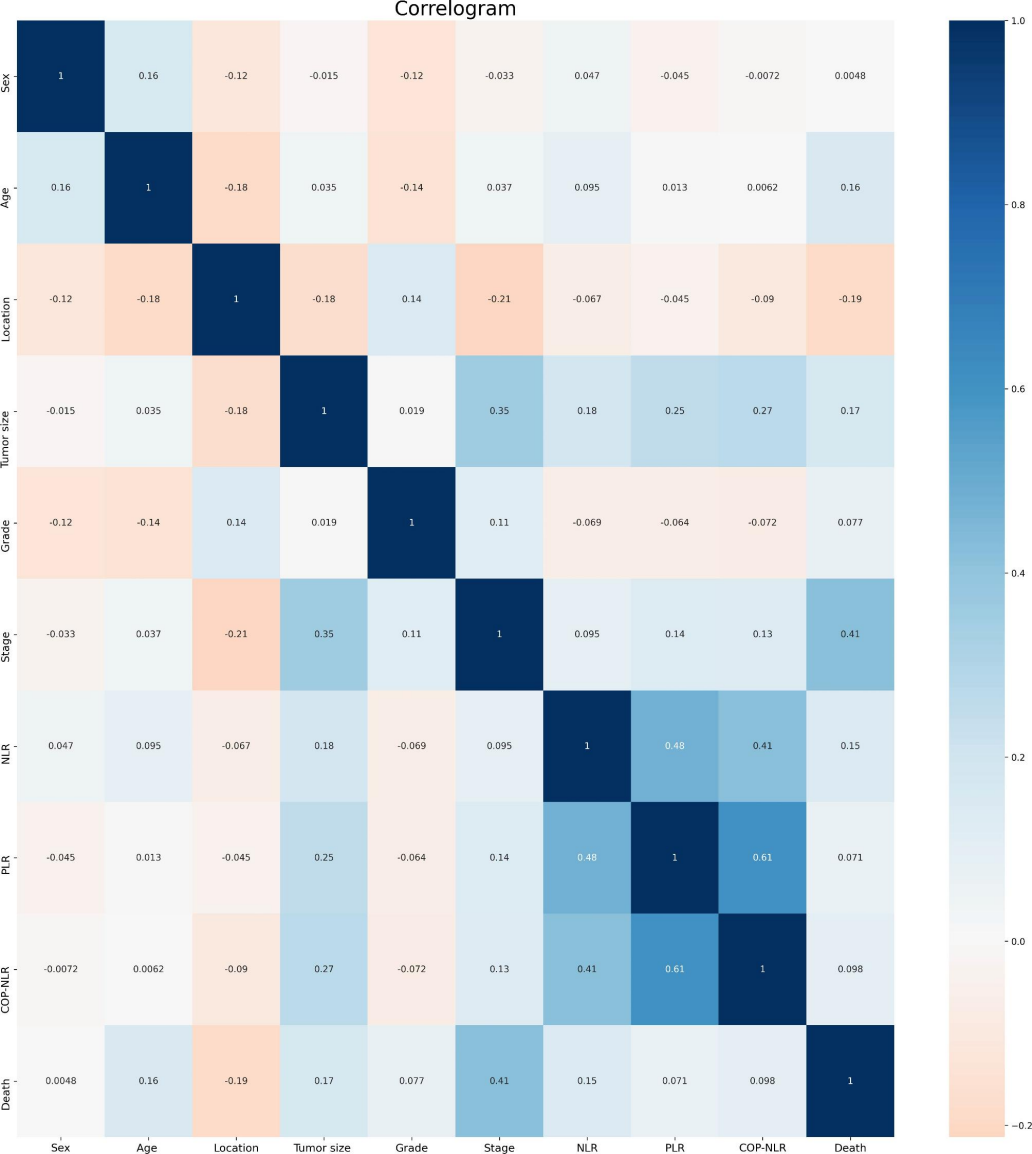
Correlation between variables

**Fig. 2:**
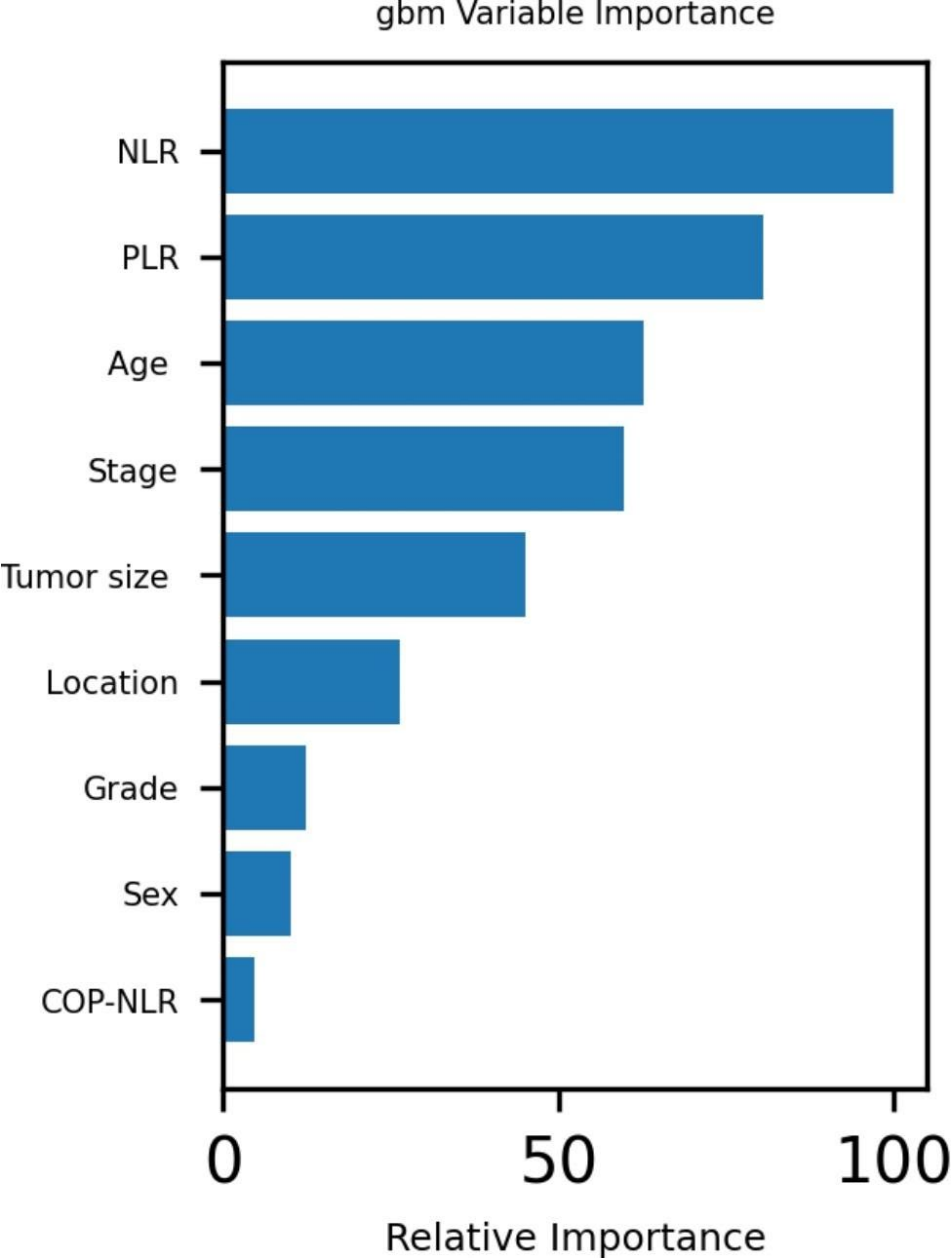
Variable importance of features included in the machine learning algorithm for predicting postoperative death outcomes for gastric cancer Note: GBM: LightGBM

The results of correlation analysis showed that age, tumor stage, NLR and PLR were positively correlated with gastric cancer among postoperative patients (see Fig. 1). In addition, the GBM algorithm factors accounting for the prognosis weight outcome show the three most important factors as NLR, PLR and age (see Fig. 2).

**Fig. 3:**
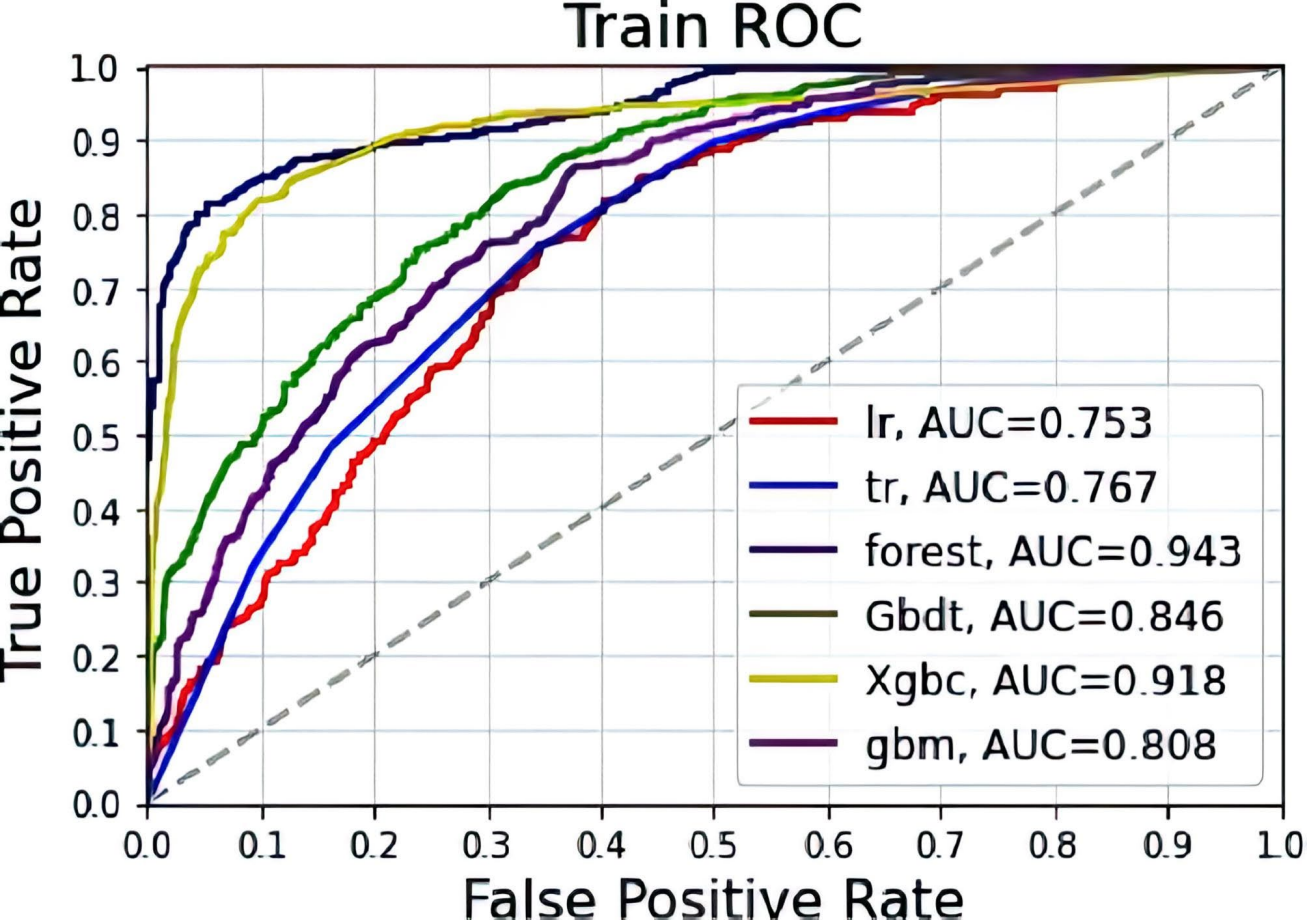
Machine learning algorithms predict gastric cancer postoperative death outcomes in the training group

Effect of the total postoperative death model in patients with gastric cancer from the predictive training group: Among the six algorithm models, forest was the most accurate (0.884), followed by Xgbc (0.868). For the six algorithms, the AUC values, from high to low, were forest, Xgbc, GBDT, GBM, Tr and LR. Among the six algorithms, forest had the highest precision and recall rate (precision = 0.876 and recall = 0.823), followed by Xgbc (precision = 0.859 and recall = 0.797) (Fig. 3 and Table 2).

**Table 2:** Forecast Results for Training Group

	Accuracy	Precision	Recall	AUC
LR	0.680	0.609	0.525	0.753
Tr	0.698	0.659	0.484	0.767
Forest	0.884	0.876	0.823	0.943
GBDT	0.756	0.675	0.730	0.846
Xgbc	0.868	0.859	0.797	0.918
GBM	0.725	0.641	0.685	0.808

Abbreviations: Logistic regression (LR); Decision tree algorithm (Tr); Random forest (Forest); Gradient Boosting algorithm (GBDT); LightGBM (GBM)

**Fig. 4:**
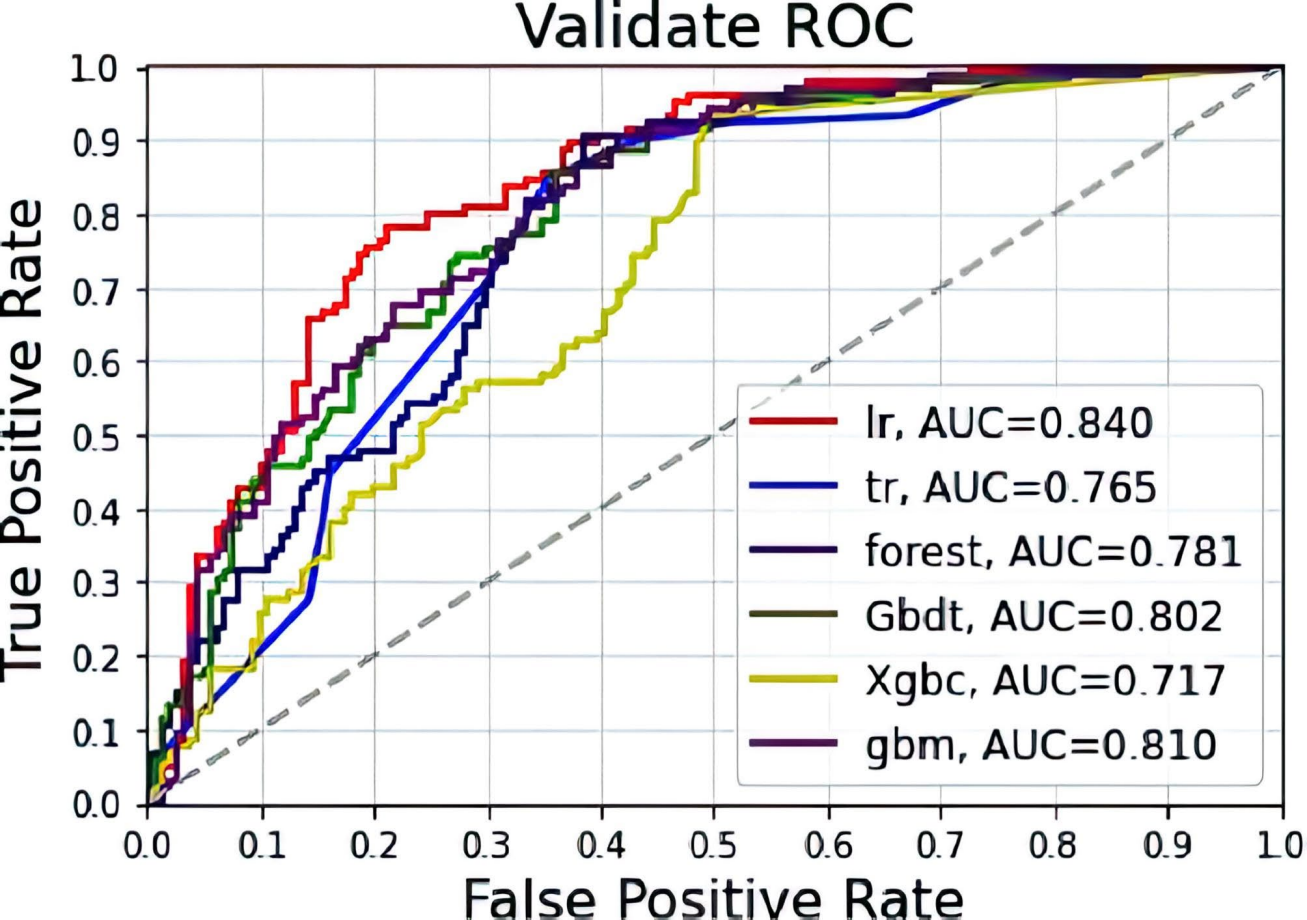
Machine learning algorithms predict gastric cancer postoperative death outcomes in the test group

Effect of the total postoperative death model in patients with gastric cancer from the predictive test group: The highest accuracy was LR (0.759), followed by the GBM algorithm (0.733). For the six algorithms, the AUC values, from high to low, were LR, GBM, GBDT, forest, Tr and Xgbc. Among the six algorithms, Logistic had the highest precision (precision = 0.736), followed by the GBM algorithm (precision = 0.660). Among the six algorithms, GBM had the highest recall rate (recall = 0.667).(Fig. 4 and Table 3).

**Table 3:** Forecast Results for Testing Group

	Accuracy	Precision	Recall	AUC
LR	0.759	0.736	0.610	0.840
Tr	0.684	0.644	0.448	0.765
Forest	0.673	0.587	0.581	0.781
GBDT	0.718	0.642	0.648	0.802
Xgbc	0.658	0.574	0.514	0.717
GBM	0.733	0.660	0.667	0.810

Abbreviations: Logistic regression (LR); Decision tree algorithm (Tr); Random forest (Forest); Gradient Boosting algorithm (GBDT); LightGBM (GBM)

## Discussion

The tumor inflammation factors NLR and PLR have a predictive effect on prognosis for a variety of tumors. Increasing NLR often indicates poor prognosis, increasing tumor stage, poor treatment response, disease-free survival and short total survival in patients with malignant tumors. However, its internal computer system is neither clear nor accurate yet [18, 19], and neither is its internal mechanism. The results of the present study show that machine learning algorithms can predict the prognosis of gastric cancer. At the same time, among these factors, the three most important factors, ranked sequentially, are NLR, PLR and age.

Jiang et al. [20] have studied the relationship between NLR and gastric cancer, and found that the NLR levels in the gastric cancer group were significantly higher than those in either the gastric polyposis group or the benign gastric tumor group. L. Lian et al. [21] studied the effect of NLR and PLR on the prognosis of gastric cancer and found that the PLR and NLR levels in patients with gastric cancer before surgery were significantly higher than those in healthy subjects. They also found that the lower PLR and NLR levels before surgery had better clinicopathological features, lower invasion depth and less lymph node metastasis. Pietrzyk et al. [22] studied 61 patients with gastric cancer and 61 healthy subjects, and found that the MPV, RDW, NLR and PLR levels in patients with gastric cancer were significantly higher than those in the control group, and that the difference was statistically significant. The present study produced similar results.

NLR predicts poor prognosis among patients with gastric cancer and it is correlated with antitumor therapy efficiency. Studies have shown that the curative effect of chemotherapy in patients with high NLR tumors is significantly lower than that in patients with low NLR tumors [23]. Studies have also shown that high NLR is correlated with an increase in PD-1 + T cells [24].

PD-L1/PD-1 pathway promotes tumor immune tolerance by preventing the inhibition effect of T-cell apoptosis. It also inhibits T-cell activation and antitumor immune response. The mechanism of antitumor therapy for PD-L1/PD-1 lies in tumor inhibition immune privilege, which increases the effect of anti-tumor immune cells [25]. In this study, inflammation-related factors predicted poor prognosis for gastric cancer after surgery. It was also found that NLR and PLR are positively correlated with tumor size. This raises several questions: can immunologic therapy solve the adverse induction effect of inflammatory-related factors on tumors? Is NLR an effective screening index for patients receiving PD-1/PD-L1 therapy?

Studies have shown that NLR and PLR are related to various clinical and pathological GC indexes, while NLR and PLR may be markers of GC disease progress. Chen X. D. et al. [26] have proposed that pre-PLRs levels are an independent predictor of peritoneal metastasis in patients with GC. When combined with various pathological features of gastric cancer (including depth of invasion, lymphoid invasion and pathological stage), the prediction results are more reliable. Kim. E. Y. et al. [27] divided 1,986 GC patients who had undergone therapeutic surgery into the following groups: high and low PLR groups and high and low NLR groups. The results of a comparison between the two groups’ clinical characteristics showed that high NLR and PLR were correlated with poor prognosis. However, NLR was a better predictor of the overall survival rate than PLR. These results are similar to the results of this study.

We are currently in the midst of a boom in health and physical therapy data. Thus, we can expect even more advances in the accuracy of artificial intelligence-assisted diagnosis and the treatment of gastric cancer in the future. Moreover, since data on gastric cancer patients are being generated at accelerating speeds and volumes, the existing PLR and NLP models cannot generate new models according to the new data, and thus the old models’ performance deteriorates due to improvements in diagnosis and treatment data for gastric cancer patients. However, artificial intelligence algorithms can dynamically learn from the collection of gastric cancer-related data. In this way, they self-learn, and gradually improve the diagnosis and prognosis of gastric cancer. Moreover, when a patient is discharged from the hospital, their diagnosis and treatment condition can be fed back to the doctor through an intelligent app, so that their recovery can be evaluated, with data stored and viewed in real-time.

Machine learning in artificial intelligence algorithms is prone to over-fitting and under-fitting in the construction of prediction models. Underfitting refers to when a model performs poorly in a training set, verification set and/or test set; over-fitting means that a model performs well in a training set, but poorly in a verification and/or testing stage, that is, the model has poor generalizability. In this study, the GBM algorithm was the most stable in both the training group and the test group. Therefore, it is the most reliable for determining the weight of various risk factors. However, multi-center queue research must be incorporated to train the model, in order to improve its performance.

This study has several limitations. Firstly, it is a retrospective study of postoperative patients with gastric cancer. And the greater bias is that there is likely a specific phenotype among East Asian patients that is different than other demographic groups which could render our ML model less useful when applied broadly, though it would still be valuable among East Asian patients, who likely have the most to gain from such a model given their higher incidence of GC. Furthermore, we have neither analyzed nor given predictions for gastric cancer patients with other malignant tumors. Finally, the variation trends for NLR and PLR in the occurrence and progression of gastric cancer could be refined and discussed further in the future. However, as NLR and PLR detection have the advantages of convenient and rapid detection, future research is needed to verify their clinical effects. Thus, a large sample study will be needed in the future. Moreover, the mechanism of the interaction between inflammation and gastric cancer needs further study. This could provide a new target for molecular targeted therapy for gastric cancer.

## Conclusion

Inflammation, as a feature of gastric cancer, provides a new direction for further study of invasion and metastasis in gastric cancer. The results of this study show that machine learning can improve the prediction of gastric cancer prognosis after surgery.

## Data Availability

Data is available at the BioStudies database (https://www.ebi.ac.uk/biostudies/europepmc/studies/S-EPMC5373584).
